# Full‐Endoscopic Approach Forchronic Low Back Pain from Baastrup's Disease: Interspinous Plasty

**DOI:** 10.1111/os.12988

**Published:** 2021-03-29

**Authors:** Wen‐tao Lin, Fa‐qin Xie, Song‐hui Lin, Ruo‐bing Yang, Huan‐wu Shen, Xue‐feng Cai, Wei Chen, Zhi‐yun Wang

**Affiliations:** ^1^ Department of Spine Surgery, Shunde Hospital Southern Medical University, The First People's Hospital of Shunde Foshan China

**Keywords:** Baastrup's disease, Full‐endoscopy, Minimally invasive surgery, Low back pain

## Abstract

The objective was to introduce a new endoscopic technique‐interspinous plasty for low back pain from Baastrup's disease; based on clinical manifestations, imaging findings and diagnostic test, to discuss a detailed diagnostic procedure for Baastrup's disease; and to explore the mechanism of interspinous plasty in pain relief. To our knowledge, there is no report about the results of endoscopic lumbar technique for Baastrup's disease. This study described the successful full‐endoscopic surgical treatment for Baastrup's disease, providing a brand‐new therapeutic method for patients. Clinical manifestations, imaging findings, including X‐ray, computed tomography (CT) and magnetic resonance imaging, and a “positive” diagnostic test with local anesthetic were used to confirm Baastrup's disease in the three included patients. The interspinous plasty procedure, which aimed to recover a physiological gap between the adjacent spinous processes, was performed by full‐endoscopic resection of marginal osteophytes. The removal of local inflamed tissue and reducing inflammation *via* intraoperative saline irrigation also lead to pain relief. Clinical outcomes included visual analog scale (VAS) for low back pain and the Oswestry Disability Index (ODI). The distance between the adjacent spinous processes was measured from the preoperative and postoperative CT scan. We calculated and recorded the difference between preoperative and postoperative measurements. Technical procedures and advantages of interspinous plasty are introduced. The three patients showed improvement in terms of the postoperative VAS of low back pain (from 8 to 2, from 7 to 1 and from 8 to 2) and ODI (from 68.9% to 33.3%, from 77.8% to 28.9% and from 71.1% to 28.9%, respectively) at the 12‐month follow‐up. Compared postoperative ODI index, the ODI index increased from 26.7% to 33.3% and from 24.4% to 28.9% in two of the cases at the 12‐month follow‐up. At 1 week, CT confirmed an obvious reduction in the marginal osteophytes between the adjacent spinous processes. Compared with those on preoperative CT images, the distance between adjacent spinous processes on postoperative CT was enlarged from 1 to 4 mm, and a repeated CT scan 3 months later reconfirmed little recrudescent osteoproliferation. In selected cases, full‐endoscopic surgical treatment for chronic mechanical back pain as part of the phenomena of Baastrup's disease may be beneficial. The operations in this study were performed under local anesthesia, with satisfactory early clinical outcomes and a low incidence of complications or adverse events. This may be a feasible therapeutic method or an alternative option for patients who cannot tolerate general anesthesia surgery.

## Graphical abstract

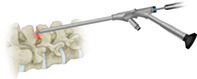

The interspinous plasty, aimed to recover a physiological gap between the spinous processes, were performed by full‐endoscopic resecting marginal osteophytes. Our study was designed to describe the successful full‐endoscopic approach for low back pain from Baastrup's disease, providing a brand‐new therapeutic method for patients.

## Introduction

Baastrup's disease was first systematically described by Baastrup in 1933. Also known as “kissing spines” and lumbar interspinous bursitis, it is characterized by hyperostosis and sclerosis between adjacent spinous processes^1^. The pathogenesis of Baastrup's disease is considered to be a lumbar excessive lordosis with resultant mechanical pressure process, which leads to repetitive strains of the interspinous ligament with subsequent degeneration and collapse, and finally to impingement between adjacent spinous processes^2^. In addition, the loss of intervertebral disc height in degenerative disc disease may also lead to closer proximity between adjacent spinous processes. As a degenerative disease, it most commonly occurs in the elderly population, with a peak incidence of 81.3% among patients older than 80 years and give rise to midline pain and tenderness^3^. Approximately 8.2% of patients who suffered from back pain or leg pain were presented with lumbar interspinous bursitis (Baastrup's disease) on magnetic resonance imaging (MRI) findings. It was most common at L_3_–L_5_ levels and was associated with older age, disc bulging, central canal stenosis and anterolisthesis^4^.

Low back pain from Baastrup's disease is implicated in mechanical contact between adjacent spinous processes, subsequently resulted in chronic interspinous inflammation. As a clinical syndrome, there are not well‐defined diagnostic criteria of Baastrup disease. In previous study, clinical diagnosis of Baastrup's disease typically relied on patients' symptoms and imaging findings. Baastrup's disease manifests clinically as a long‐term medical history of localized midline lumbar pain, which can be aggravated by lumbar extension and relieved by lumbar flexion.

Characteristic imaging findings are usually presented with the “kissing” of closely approximated spinous processes and sclerosis of the articulating surfaces in standard lateral X‐ray. Computed tomography (CT) axial images and sagittal and coronal reconstructions show in detail that sclerosis, flattening and enlargement of the two adjacent spinous processes surfaces^3^. MRI findings reveal the presence of interspinous bursitis in interspinous region which may precede the more pronounced osseous changes of the spinous processes. Baastrup's disease can exist independently or together with other spine related disorders^4^. In the differential diagnosis, it is important to exclude other sources of chronic low back pain, including lumbar discogenic pain, facet joint syndrome, Bertolotti's syndrome and sacroiliac joint pain^5^. Baastrup's disease should be considered when low back pain is aggravated by extension and alleviated by flexion. Both dynamic imaging and local anesthetic injection can assist the clinician in making a diagnosis.

However, Baastrup's disease is frequently underdiagnosed and often missed owing to lack of knowledge of the disease process or improper diagnostic techniques. As a result, it may lead to inappropriate treatment, treatment delay, even no treatment^6^. Numerous clinical treatments exist, mainly including oral or topical medication, local injections of glucocorticoid and surgical excision of the spinous process, but this is an ongoing topic of debate. Poor long‐term results of conservative treatment are the main limiting factor, and traditional surgical excision of the spinous process has shortcomings including large surgical trauma, destruction of spinal stability, and uncertain clinical efficacy. In a study of 55 patients with Baastrup s disease, 22% of patients required a second infiltration within 7–10 days after first injection of corticosteroid with local anesthetic^7^. Another study reported that only 11 in 64 patients with Baastrup's disease had a long‐term efficacy after partial or total excision of the pathological spinous processes^8^. The optimal clinical decision regarding treatment remains unresolved.

With the rapid development of spinal endoscopic techniques in recent years, full‐endoscopic procedures represent a method of surgical treatment with a smaller “surgical footprint” to treat spinal pathology, such as lumbar disc herniation, lumbar spinal stenosis, and lumbar spondylolisthesis^9^. There are several technical advantages often cited to support the widespread application of full endoscopic spinal surgery. Continuous retraction of the soft tissue and dissection of the paraspinal muscles with resultant necrosis during traditional open procedures have been considered as a cause for worse clinical outcomes^10^. Compared to traditional open procedures, full‐endoscopic technique is characterized by less soft tissue damage and blood loss, less postoperative back pain, smaller and cosmetically more acceptable incisions, shorter length of hospital stays^11^, ^12^, ^13^. Additionally, under high visual magnification, surgical operations can be performed more accurately and efficiently using full‐endoscopic technique. Therefore, full‐endoscopic surgery may serve as a brand‐new and effective therapeutic method for chronic back pain from Baastrup's disease. In this context, we envision that endoscopic resection of the partial pathological spinous process may restore physiological and anatomical condition in interspinous region, which reduce the risk of impingement between the adjacent spinous processes. To the best of our knowledge, our study is the first to report a successful full‐endoscopic approach for Baastrup's disease, termed interspinous plasty, with satisfactory clinical outcomes.

Specific aims of our study are: (i) to introduce a new endoscopic technique‐interspinous plasty; (ii) to discuss a detailed diagnostic procedure of Baastrup's disease; and (iii) to explore the mechanism of interspinous plasty in pain relief.

## Materials and Methods

### Inclusion and Exclusion Criteria

Inclusion criteria were as follows: (i) diagnosis of Baastrup's disease; (ii) interspinous plasty; (iii) persistent low back pain and conservative measures did not work‐well; (iv) evaluation of pain relief; and (v) case series. Exclusion criteria were as follows: (i) less than 18 years old; (ii) patients experienced radicular leg pain or neurogenic intermittent claudication; and (iii) history of other lumbar surgery.

### Patients

**Fig. 1:**
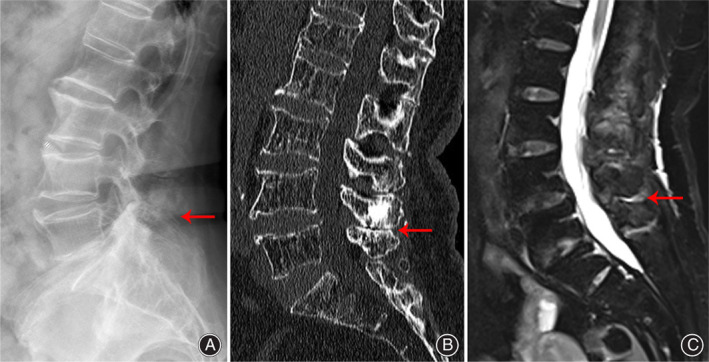
The preoperative imaging data of the patient. (A) X‐ray showed apposition of the dorsal spinous processes. (B) CT showed severe hyperostosis and osteosclerosis on the edges of the L_4_ and L_5_ spinous processes. (C) MRI showed a high signal on fat‐suppressed T2‐weighted images around the L_4_ and L_5_ spinous processes.

Three patients who experienced recurrent mechanical back pain for more than 1 year, but without associated leg pain were included in this study. Back pain increased with extension, was relieved by flexion and improved only minimally with nonsteroidal anti‐inflammatory drugs (celecoxib and topical flurbiprofen plaster) and physical treatments (traditional Chinese medicine packets, acupuncture). The focal midline back pain was directly reproduced by palpation over the spinous processes, but no tenderness was observed upon palpation of the lumbar facet joints. Profile views of X‐ray examinations at upright neutral, flexion, and extension positions were performed. Hyperostosis and osteosclerosis of the spinous processes were evaluated by CT. MRI was performed to evaluate edema and inflammation of the interspinous ligaments in fat‐suppressed T2‐weighted images (Fig. 1). Details of the patients' clinical characteristics and pain scores are shown in Table 1.

**TABLE 1:** Clinical characteristic of the study participants

Patients	Age (years)	Gender	Back pain (duration, years)	Pre‐op VAS	Pre‐op ODI (%)	X‐ray (contact of SP)	CT (hyperostosis and osteosclerosis of SP)	MRI (IL on fat‐suppressed)	Level
1	68	Female	7	8	68.9	Extension position	Severe	T2 high	L_4_/L_5_
2	76	Female	5	7	77.8	Upright neutral position	Moderate	T2 high	L_3_/L_4_ L_4_/L_5_
3	66	Male	1	8	71.1	Extension position	Moderate	T2 high	L_4_/L_5_ L_5_S_1_

IL, interspinous ligaments; ODI, oswestry disability index; SP, spinous process; VAS, visual analog scale.

### Diagnostic Test

**Fig. 2:**
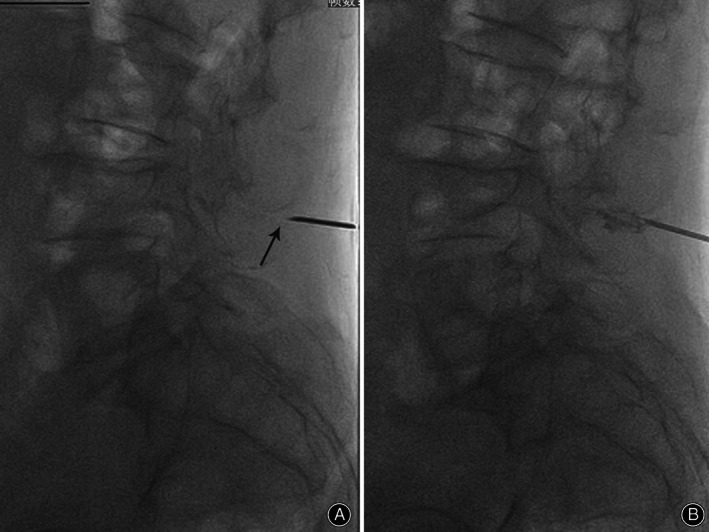
An illustration of lidocaine injection. (A) Lateral view of a needle tip (arrow) placed in the interspinous area (L_3‐4_, patient 2). (B) The mixture of lidocaine with contrast media injected through the needle, and contrast infiltration can be seen within the interspinous area.

The diagnoses of the three patients were confirmed by the relief of pain after fluoroscopy‐guided injection of local anesthetic (2 mL of 2% lidocaine) into the interspinous ligament (Fig. 2). The patients' low back pain was examined again 10 min after the first injection, and the VAS scores were compared with those before the injection. The diagnostic test was defined as “positive” if the patients indicated less than 60% of their preoperative VAS scores at 10 min; otherwise, it was “negative”^14^ (Table 2).

**TABLE 2:** Evaluation of low back pain after interspinous lidocaine injection

Patients	Pre‐injection VAS	Post‐injection VAS	Pain relief (%)	Diagnostic test
1	8	3	62.5	Positive
2	7	2	71.4	Positive
3	8	2	75.0	Positive

VAS, visual analog scale.

### Surgical Technique

#### Establish Endoscopic Working Channel

The interspinous plasty for case 1 was performed with the patient in the prone position under local anesthesia with 5 mL of 2% lidocaine mixed with 5 mL of normal saline. A blood pressure cuff, pulse oximeter and automatic electrocardiogram were utilized for monitoring. Based on anteroposterior and lateral C‐arm guidance, the surgeon inserted a needle with a 20 mL sterile syringe into the L_4‐5_ interspinous ligament as an endoscopic positioning mark. A 15 cm, 18‐gauge needle was inserted obliquely into the interspinous ligament of L_4‐5_ approximately 5 cm from the midline, and then a guide wire was introduced through the needle. Thereafter, the 18‐gauge needle was removed, and a tapered obturator was slid into the interspinous area along the guide wire. Finally, a working cannula (7 mm diameter) with a bevel was docked into the interspinous area and reconfirmed by C‐arm fluoroscopy.

#### Enlargement of Interspinous Gap by Full Endoscopic Procedure

**Fig. 3:**
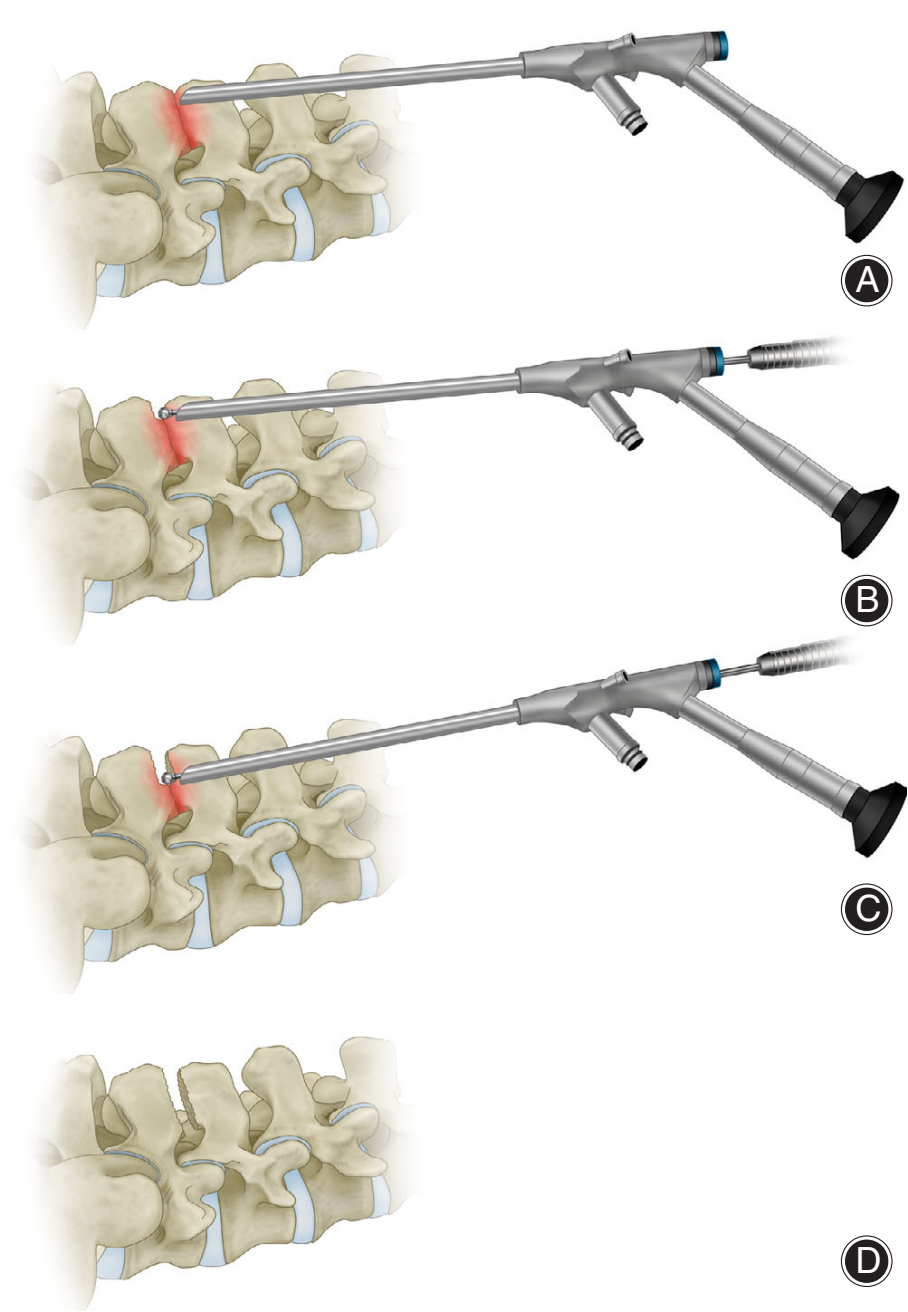
The diagram of interspinous plasty. (A) A working endoscopy is placed at the top of contact surfaces between adjacent spinous processes; (B, C) Through an endoscopic working cannula and using a high‐speed drill, an excision procedure for spinous processes was performed from dorsal to ventral; (D) A enlarged interspinous gap after interspinous plasty.

Under high visual magnification, the adjacent spinous processes collided tightly with almost no gap. Through an endoscopic working cannula and using a high‐speed drill, the surgeon performed an excision procedure of the inferior margin of the L_4_ and the superior margin of the L_5_ spinous processes until the ventral ligamentum flavum was exposed and adequate space was obtained. Significant, excessive extrusion of the ligamentum flavum can compress the nerve root and produce iatrogenic nerve root injury. After excision of the tissue, adequate hemostasis was performed on the internal tissue wound by plasma radiofrequency, and the incision was sewn after withdrawing the endoscopic working cannula (Fig. 3).

### Clinical Assessment

#### Visual Analog Scale (VAS)

Low back pain was evaluated using VAS preoperatively, after interspinous injection of lidocaine, after interspinous plasty and at the postoperative follow‐up. The VAS scoring system was a continuous single scale item, which refers to a ruler about 10 cm long, marked with 10 scales on one side. A score of “0” indicates no pain, and a score of “10” indicates the most severe pain. According to the clinical evaluation, “1–3” was classified as mild pain, “4–6” as moderate pain, “7–10” as severe pain.

#### Oswestry Disability Index (ODI)

Overall condition in quality of life was assessed using ODI preoperatively, after interspinous plasty and at the postoperative follow‐up. The ODI score system includes 10 sections: pain intensity, personal care, lifting, walking, sitting, standing, sleeping, sex life, social life and traveling. For each section of six statements the total score is 5. Intervening statements are scored according to rank. If more than one box is marked in each section, take the highest score. If all 10 sections are completed the score is calculated as follows: total scored out of total possible score × 100. If one section is missed (or not applicable) the score is calculated: (total score/(5 × number of questions answered)) × 100%. From 0% to 20% is considered mild dysfunction, 21%–40% is moderate dysfunction, 41%–60% is severe dysfunction, and 61%–80% is considered as disability. For cases with score of 81%–100%, either long‐term bedridden, or exaggerating the impact of pain on their life.

### Radiographic Assessment

All patients underwent the CT scanner using the 64‐slice spiral CT machine (Aquilion PRIME; Toshiba Medical Systems Co., Japan). The following parameter was measured on the CT radiographs: interspinous gap (the distance between the inferior margin of the superior spinous process and the upper margin of the inferior spinous process). An investigator measured the parameter three times, calculated and recorded its average. The interspinous gap was measured again from the postoperative CT scan. Preoperative and postoperative interspinous gaps were comparatively analyzed.

## Results

Table 1 showed the average age of the included patients was 70, and the average preoperative VAS and preoperative ODI of the included patients were 7.7%, 72.6%, respectively. All patients had visible mechanical contact of the spinous processes in extension or upright position X‐rays. CT scans revealed moderate or severe hyperostosis and osteosclerosis of the spinous processes, and MRI findings showed a high signal between interspinous ligaments on fat‐suppressed T2‐weighted images. On diagnostic test, the three patients performed positive for interspinous lidocaine injection (Table 2).

### Clinical Outcome

The three patients showed improvement in terms of the postoperative VAS of low back pain (from 8 to 2, from 7 to 1 and from 8 to 2) and ODI (from 68.9% to 33.3%, from 77.8% to 28.9% and from 71.1% to 28.9%, respectively) at the 12‐month follow‐up. Compared postoperative ODI index, the ODI index increased from 26.7% to 33.3% and from 24.4% to 28.9% in two of the cases at the 12‐month follow‐up (Table 3).

**TABLE 3:** The clinical outcome of the study participants at the 12‐month follow‐up

Patients	Pre‐op VAS	Post‐op VAS	12‐month follow‐up VAS	Pre‐op ODI (%)	Post‐op ODI (%)	12‐month follow‐up ODI (%)	Complications
1	8	1	2	68.9	26.7	33.3	None
2	7	1	1	77.8	28.9	28.9	None
3	8	2	2	71.1	24.4	28.9	None

ODI, oswestry disability index; VAS, visual analog scale.

### Radiographic Outcome

**Fig. 4:**
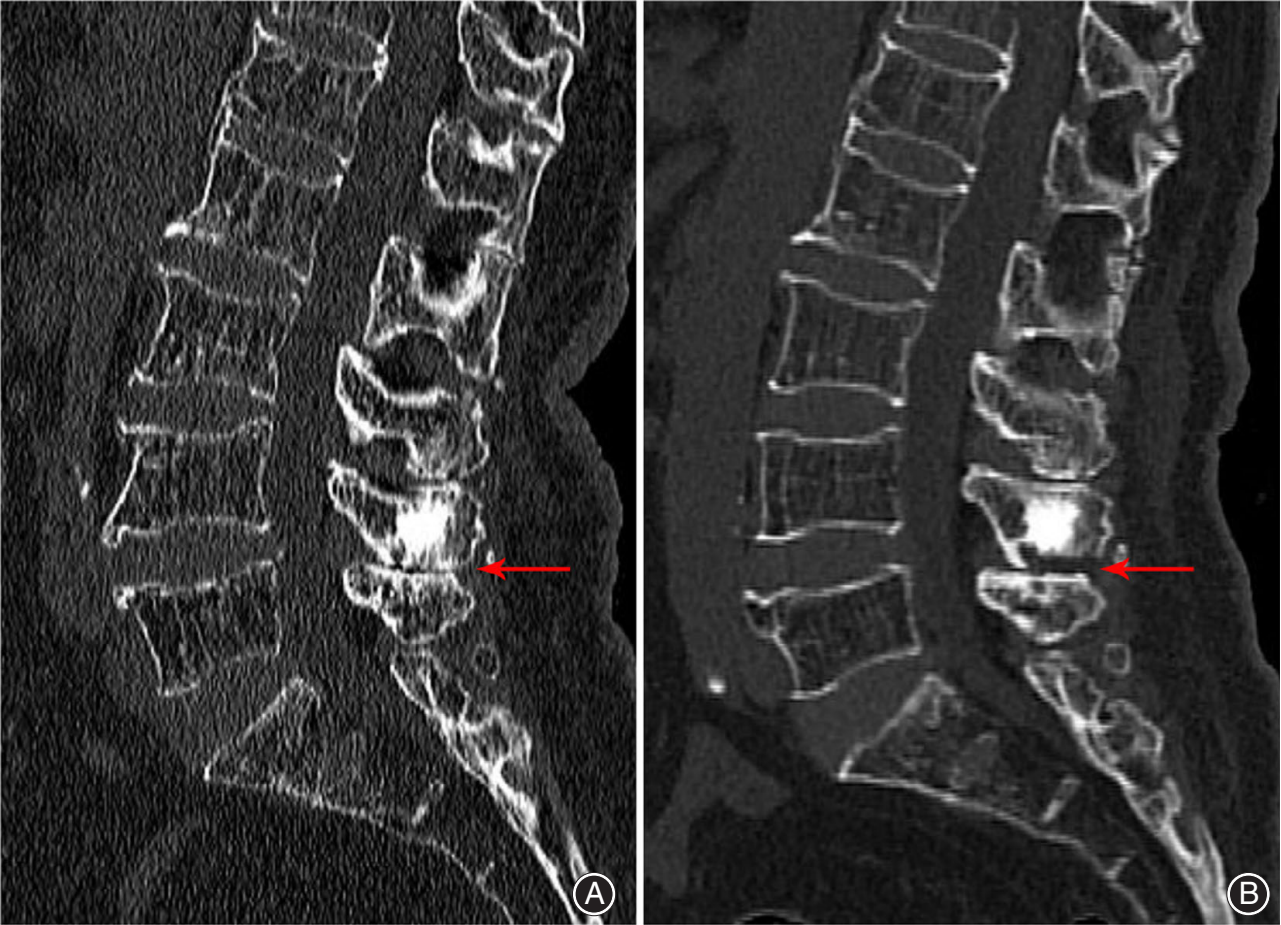
Compared with the preoperative CT images (A), the postoperative CT (B) showed that the distance between the L_4_ and L_5_ spinous processes had been enlarged from 1 mm to 4 mm.

At 1 week, computedtomography (CT) confirmed an obvious reduction in marginal osteophyte between the adjacent spinous processes. Compared with those on preoperative CT images, the distance between the adjacent spinous processes on postoperative CT was enlarged from 1 to 4 mm (Fig. 4), and repeated CT scan 3 months later reconfirmed little recrudescent osteoproliferation.

## Discussion

Chronic low back pain can lead to physical and psychological discomfort and negative effects on one's health and quality of life^15^. Most patients previously reported in the literature were managed with various treatment for chronic low back pain from Baastrup's disease, including conservative management, fluoroscopy‐guided glucocorticoid injections and traditional open surgery. To the best of our knowledge, this is the first report showing clinical improvement with a full‐endoscopic technique for Baastrup's disease.

### Surgical Technique and Advantages of Interspinous Plasty

**Fig. 5:**
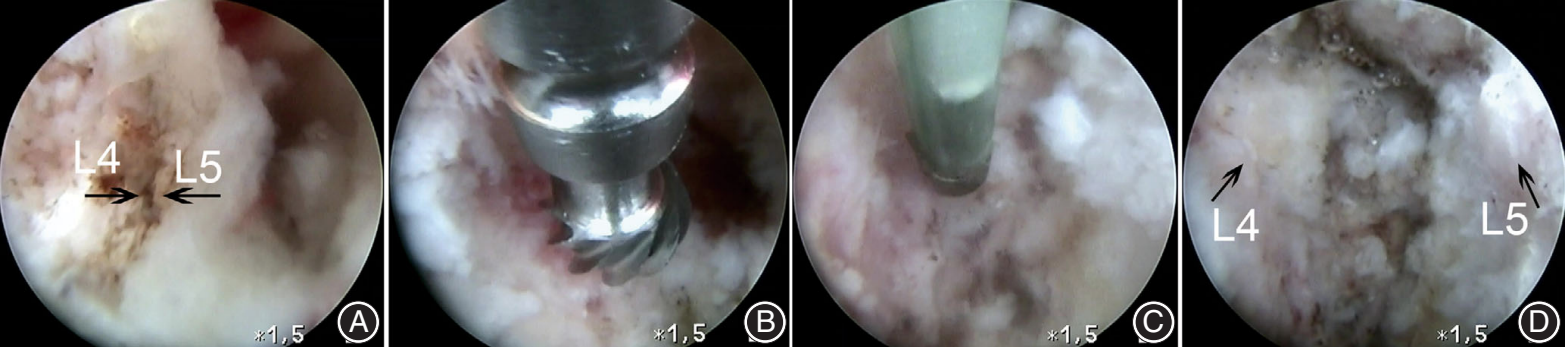
An introduction of surgical technique. (A) Mechanical contact between L_4_ (left arrow) and L_5_ (right arrow) spinous processes; (B) Excision procedure of partial spinous processes by using a high‐speed drill; (C) Endoscopic hemostasis by plasma radiofrequency. (D) The distance between L_4_ (left arrow) and L_5_ (right arrow) increased after interspinous plasty.

Lumbar full‐endoscopic procedures have already become common surgical methods for spinal diseases such as lumbar disc herniation and spinal stenosis, with the clear advantages of safety and effectiveness^16^, ^17^, ^18^. Interspinous plasty aims to restore physiological and anatomical condition between the adjacent spinous processes by removing marginal osteophytes and local inflammatory tissues. In our study, partial surgical excision of the lumbar spinous processes with hyperplasia and sclerosis was performed through a 7 mm surgical incision assisted by a working channel. The interspinous space was enlarged, which would prevent the spinous processes from hitting each other during extension (Fig. 5). Minimal internal tissue injury and a small surgical incision (approximately 7 mm) resulted in a short recovery period, minimal scar tissue formation, and few complications. Less intraoperative blood loss encouraged fast wound healing, reduced the risk of infection and accelerated perioperative care and rehabilitation. Furthermore, preservation of the majority of the spinous processes and the supraspinous ligament contributed to maintenance of spinal stability and a reduction in the back pain caused by unstable spines^19^.

### Clinical and Radiographic Outcomes after Interspinous Plasty

During the 12‐month follow‐up, the ODI index increased from 26.7% to 33.3% and from 24.4% to 28.9% in two of the cases (Table 3). In our study, considering the effect of psychological or psychosocial factors, a consideration of sexual function was excluded. The abovementioned two cases had 0 points after operation for section 4 (walking), but this increased to 2 points at the 12‐month telephone follow‐up. The score of the former case increased from 1 to 2 points in section 5 (sitting) at the 12‐month follow‐up. Based on the patients' real outcome, the increased ODI indexes were reported, which may have resulted from knee osteoarthrosis and degenerative spine‐related disease. The long‐term clinical outcomes deserve further study.

One patient showed an improved VAS score for low back pain and an improved ODI after the operation and at the 12‐month follow‐up, but the postoperative CT showed that the L_4‐5_ spinous process space was widened on the posterior side and still narrow at the base (Fig. 4). Impingement between the adjacent spinous processes was a major cause of low back pain. However, the remaining bone spur of the L_4_ spinous process was not the collision point at the collision moment. The key collision point, caused chronic low back pain, was located in the posterior side of L_4‐5_ spinous processes space, which had been enlarged by full‐endoscopic partial excision of the L_4‐5_ spinous processes. Local inflammatory responses to long‐term L_4‐5_ spinous process impingement was also a cause of chronic pain. The mechanism of pain relief by interspinous plasty may be associated with the surgical enlargement of the interspinous space and the removal of local inflamed tissue, as well as by reducing inflammation *via* intraoperative saline irrigation.

### Development of Baastrup's Disease Treatment

Singla *et al*.^20^ reported a patient who was managed conservatively with muscle relaxants and analgesics for 1 week and then started physiotherapy with spinal flexion exercises at regular intervals. At the 6‐month follow‐up, the improvement in back pain and mobility was durable. Larger sample sizes and an assessment of long‐term outcomes should be considered in the future.

In 2007, Mitra *et al*.^21^ first presented a simple case report of an 89‐year‐old patient who underwent a fluoroscopy‐guided interspinous injection of triamcinolone acetate under local anesthetic. Lamer *et al*.^22^ reported that fluoroscopy‐guided injections provided pain relief in all three patients in a 2008 study. Two of them had a long‐term response to local anesthesia and corticosteroid injections (2–3 mL of 0.25% bupivacaine and 3 mg of betamethasone), and one patient eventually underwent resection of the involved spinous processes on account of recurrent pain. Two prospective studies also demonstrated comparable clinical improvement following fluoroscopy‐guided infiltration in patients with Baastrup's disease. One study of 17 patients by Okada *et al*.^14^ in 2014 revealed that patients who underwent injection of local anesthetic and steroid into the interspinous ligaments for Baastrup's disease experienced pain relief. A later prospective study by Filippiadis *et al*.^7^ conducted in 2015 was one of the largest patient samples concerning Baastrup's disease and infiltrations. This study reported a significant pain reduction and mobility improvement at the 12‐month follow‐up. Twelve patients included in the study showed poor pain relief, and a second infiltration was performed 7–10 days after the first. Glucocorticoid infiltration has been typically used to relieve pain in a variety of musculoskeletal disorders with chronic inflammation, including degenerative spine disease, osteoarthritis, and tendinopathy^23^, ^24^.

There is conflicting evidence regarding the clinical efficacy of glucocorticoid infiltration. In general, glucocorticoids have some short‐term benefit for pain relief but no significant long‐term benefit^25^. The mechanism of the low back pain caused by Baastrup's disease involves a decrease in the distance between adjacent spinous processes with opposing surfaces coming into contact. This contact causes further collisions, sclerosis, and cyst formation at the opposing surfaces along with inflammation. The short‐term efficacy of glucocorticoids may be because they decrease the chronic interspinous inflammation. However, the pathological, anatomical stenosis between the adjacent spinous processes remains intact, thus, the long‐term efficacy and recurrence rate seen with glucocorticoid infiltration should be further investigated.

Traditional surgical intervention includes excision of the intraspinous bursa and partial or total removal of the spinous process. Gold and Jang *et al*.^26^, ^27^ revealed a posterior epidural cyst and fibrotic mass associated with Baastrup's disease, resulting in neurogenic claudication that resolved after total excision. However, the effect of surgical intervention is controversial, as some of these operations do not alleviate simple back pain. Two clinical studies have demonstrated inconsistent levels of clinical improvement following traditional operations. In 1944, Franok^28^ performed an earlier study of 10 patients that showed improvement for Baastrup's disease after excision of the spinous process. In contrast, a later study by Beks *et al*.^8^ in 1989, in which 64 patients underwent either partial or total surgical excision of the spinous processes, demonstrated that only 11 patients were free of symptoms after the operation and stayed asymptomatic. Analysis of their study suggested that Baastrup's disease was seldom the disease entity but rather that another pathological phenomenon was often responsible for the back pain. It is crucial to make differential diagnoses of low back pain among spine‐related diseases, including lumbar muscle strain, spinal instability, and degenerative disc disease.

In our study, the patients suffered from a history of mechanical low back pain that increased with extension and was relieved by flexion, without radicular pain or intermittent claudication. The X‐ray images of our patients were characterized by contact between adjacent lumbar spinous processes, known as “kissing spine.” CT showed enlargement, hyperostosis and osteosclerosis between adjacent spinous processes leading to narrow interspinous gaps. MRI showed local inflammation and an edematous zone between the adjacent spinous processes on a fat‐suppressed T2‐weighted sequence. Given the abovementioned clinical manifestations and imaging findings, the patients were finally confirmed to have Baastrup's disease after a diagnostic test with local anesthetic was “positive.” The patients performed an immediate pain relief after the injection of local anesthetic, but localized back pain aggravated gradually in the next 48 h. After failed the conservative treatment, these appropriate patients were scheduled for interspinous plasty.

The spinous processes, interspinous ligament, and part of the posterior ligamentous complex play a role in spinal stability^29^. After spinal surgery, osseous continuity between the spinous processes and the lamina is important for maintaining the long‐term benefit^30^. Traditional surgical resection of the total spinous process would have damaged the mechanical stability and resulted in accelerated degeneration, which would then increase the occurrence of spine‐related disease. Interspinous plasty aims to recover a physiological gap between the spinous processes by resecting marginal osteophytes. We used a full‐endoscopic technique to remove the edges of the spinous processes (approximately 2 mm) and the local aseptic inflammatory tissues while preserving as much interspinous ligament and supraspinous ligament as possible, with little effect on spinal stability.

**Fig. 6:**
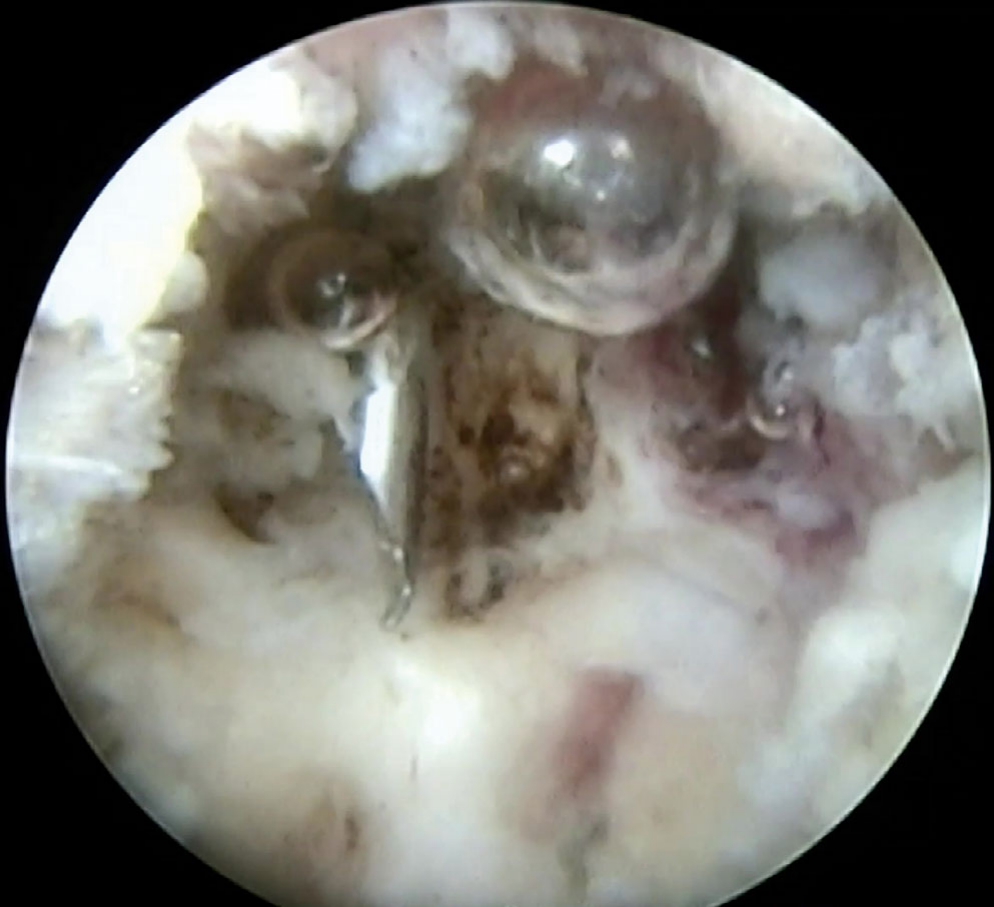
A positioning needle inserted into the interspinous space (L_4‐5_).

C‐arm fluoroscopy is an indispensable part of the endoscopic spine procedure that enhances surgical accuracy. The surgeon's exposure to ionizing radiation is harmful and is easily overlooked^31^. Given the measurable lifetime radiation hazards to the surgeon, we inserted a needle intraoperatively into the interspinous area of L_4‐5_ to reduce exposure (Fig. 6). The ability to visualize the needle under the endoscopic field of vision means reaching the expected, ideal position without the need for repeated fluoroscopy. The use of the time‐distance‐shielding principle (minimizing time, maximizing distance, and using shielding) can also reduce surgeon radiation exposure^32^. Some researchers have proposed that ultrasound‐assisted endoscopic lumbar surgery may also be a feasible way to reduce radiation exposure^33^.

### Limitations and Future Research

Undeniably, first, the small sample size and short follow‐up were considered as a major limitation of the presented study. Second, there is no comparison group of other treatment options. Although the patients in this study achieved a good functional recovery, long‐term outcomes are unknown. Moreover, we are unable to draw a convincing conclusion that interspinous plasty is better than other treatments. Compared with spinal fusion, interspinous plasty performed lesser damage to spine motion. The full‐endoscopic approach for Baastrup's disease is a promising technique with its advantages of precision and being a minimally invasive procedure. However, further comparative studies need to be performed to evaluate the efficacy and safety of this method.

### Conclusion

Various techniques have been proposed to alleviate the mechanical low back pain associated with Baastrup's disease. The full‐endoscopic technique has the advantages of minimal trauma and radiation exposure, rapid recovery and a limited negative effect on spinal stability. This technique was performed under local anesthesia, with satisfactory early clinical outcomes, and avoided the excessive iatrogenic trauma of traditional open operations. In appropriate cases, the full‐endoscopic approach for chronic mechanical back pain as part of the phenomena of Baastrup's disease may be a feasible therapeutic method.
